# Conformational Response of Influenza A M2 Transmembrane Domain to Amantadine Drug Binding at Low pH (pH 5.5)

**DOI:** 10.3389/fphys.2016.00317

**Published:** 2016-07-29

**Authors:** Elka R. Georgieva, Peter P. Borbat, Kirill Grushin, Svetla Stoilova-McPhie, Nichita J. Kulkarni, Zhichun Liang, Jack H. Freed

**Affiliations:** ^1^Department of Chemistry and Chemical Biology, Cornell UniversityIthaca, NY, USA; ^2^National Biomedical Center for Advanced ESR TechnologyIthaca, NY, USA; ^3^Department of Neuroscience and Cell Biology, Sealy Center for Structural Biology and Molecular Biophysics, University of Texas Medical Branch at GalvestonGalveston, TX, USA; ^4^College of Art and Sciences, Cornell UniversityIthaca, NY, USA

**Keywords:** influenza M2 proton channel, amantadine, protein-drug interactions, proton channel inhibition, DEER spectroscopy, ESR, protein-membrane interactions

## Abstract

The M2 protein from influenza A plays important roles in its viral cycle. It contains a single transmembrane helix, which oligomerizes into a homotetrameric proton channel that conducts in the low-pH environment of the host-cell endosome and Golgi apparatus, leading to virion uncoating at an early stage of infection. We studied conformational rearrangements that occur in the M2 core transmembrane domain residing on the lipid bilayer, flanked by juxtamembrane residues (M2TMD_21−49_ fragment), upon its interaction with amantadine drug at pH 5.5 when M2 is conductive. We also tested the role of specific mutation and lipid chain length. Electron spin resonance (ESR) spectroscopy and electron microscopy were applied to M2TMD_21−49_, labeled at the residue L46C with either nitroxide spin-label or Nanogold® reagent, respectively. Electron microscopy confirmed that M2TMD_21−49_ reconstituted into DOPC/POPS at 1:10,000 peptide-to-lipid molar ratio (P/L) either with or without amantadine, is an admixture of monomers, dimers, and tetramers, confirming our model based on a dimer intermediate in the assembly of M2TMD_21−49_. As reported by double electron-electron resonance (DEER), in DOPC/POPS membranes amantadine shifts oligomer equilibrium to favor tetramers, as evidenced by an increase in DEER modulation depth for P/L's ranging from 1:18,000 to 1:160. Furthermore, amantadine binding shortens the inter-spin distances (for nitroxide labels) by 5–8 Å, indicating drug induced channel closure on the C-terminal side. No such effect was observed for the thinner membrane of DLPC/DLPS, emphasizing the role of bilayer thickness. The analysis of continuous wave (cw) ESR spectra of spin-labeled L46C residue provides additional support to a more compact helix bundle in amantadine-bound M2TMD _21−49_ through increased motional ordering. In contrast to wild-type M2TMD_21−49_, the amantadine-bound form does not exhibit noticeable conformational changes in the case of G34A mutation found in certain drug-resistant influenza strains. Thus, the inhibited M2TMD_21−49_ channel is a stable tetramer with a closed C-terminal exit pore. This work is aimed at contributing to the development of structure-based anti-influenza pharmaceuticals.

## Introduction

**Figure 1:**
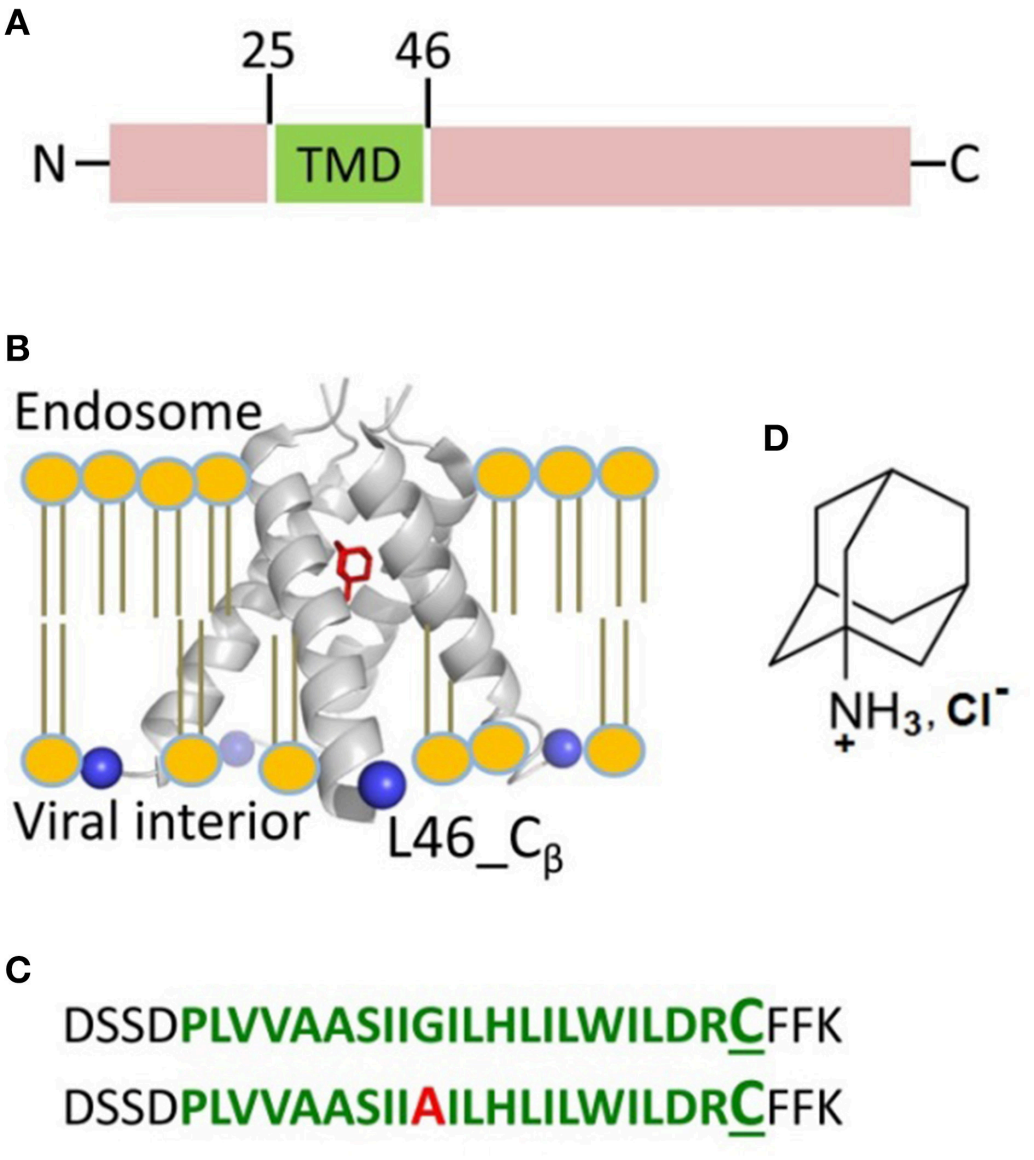
**Influenza A M2 protein. (A)** The domain organization of full-length M2 encompasses the transmembrane domain (TMD) comprised of residues 25–46 shown in green. **(B)** The structure of M2TMD tetramer reported by X-ray crystallography (PDB 3C9J): The TM helices are further away at the C-terminus; Amantadine (in red) is bound to the central pore of M2TMD channel. The C_β_-atoms of residue L46, which was substituted for cysteine in this work, are depicted as blue spheres. The lipid bilayer was added for clarity. **(C)** M2TMD_21−49_ peptides (wt and G34A mutant) used in this work: The residues corresponding to the TMD are in green, whereas the N- and C-terminal juxtamembrane residues are in black. In the second peptide, the alanine residue at position 34 is in red. The L46C residue used for spin-labeling is in enlarged font. **(D)** The amantadine hydrochloride molecule.

Influenza pandemics cause serious health concerns for humans and for depletion of livestock. To control and suppress influenza infections, a good understanding of the fundamental mechanisms underlying the structure and function of proteins that play critical roles in the viral cycle is needed. In influenza A virus one such essential protein is M2, which is a small 97 amino acid integral membrane protein containing a single transmembrane helix (Figure 1A). In lipid membranes, M2 monomers oligomerize with their transmembrane parts forming a four-helix bundle (Figure 1B). The tetramer exhibits proton channel activity (Lamb et al., 1985; Sugrue and Hay, 1991). After virus internalization, M2 activates at the low pH (5 to 6) of the host cell endosome, resulting in a proton influx that acidifies the viral interior leading to uncoating of the virion and release of the viral RNAs. In the process of replication, the M2 channel function is to support the transport of newly synthesized viral proteins through the Golgi apparatus by equilibrating the low intra-Golgi pH with that of the cytoplasm. The functional proton channel unit is a tetramer of M2 transmembrane domains (M2TMD) containing residues 22–46 (Ma et al., 2009). Two residues H37 and W41 are known to provide a unidirectional proton current; H37 shuttles the proton through the channel by altering its protonation state, whereas W41 serves as the channel gate (Okada et al., 2001; Wang et al., 2011).

Because of its role in virus proliferation, M2 is a target for drug development. Several pharmaceutical products have been developed to inhibit the M2 channel activity and more are under development. Amantadine (Figure 1D) and rimantadine are the best-studied M2 channel blockers (Hay et al., 1985; Jing et al., 2008). It is essential to understand the modes of interaction of these drugs with M2 that lead to inactivation. It is currently accepted that both amantadine and rimantadine bind to the central pore of the M2 transmembrane domain (M2TMD) obstructing the access to the H37 selectivity filter (Jing et al., 2008; Wang et al., 2011). Increased binding affinity of amantadine at neutral or basic pH, compared to acidic pH, was attributed to the change in the protonation state of H37, suggesting that this residue participates in drug binding (Salom et al., 2000). Most of the atomic-level structural studies on M2TMD-drug complexes are at basic pH (Hu et al., 2007; Cady et al., 2010), which do not represent the acidic environment of the endosome and the active state of the M2 channel. The crystal structure of amantadine-bound M2TMD tetramer at low pH 5.3 and in detergent micelles provides insights into how the drug might inhibit the channel function in low pH environment (Stouffer et al., 2008). However, the structural information on drug-free M2TMD at low pH is very limited and it is unclear how the inhibition of the M2 channel under these conditions may occur. Moreover, one could reasonably expect that the actual inhibition mechanism in a lipid bilayer could be very different from what is seen in detergent.

In this study, we sought to gain insights into the structural basis underlying inhibition of the M2 proton channel. Particularly, our focus is on the M2 oligomeric state and the conformational response of the C-terminal pore of M2TMD to amantadine drug binding at low pH (pH 5.5). To this end, we applied double electron-electron resonance (DEER) spectroscopy (Milov et al., 1984a; Borbat and Freed, 2007, 2014; Pannier et al., 2011; Jeschke, 2012) to the spin-labeled peptide containing M2TMD and its N- and C-terminal flanking residues—M2TMD_21−49_ (Figure 1C). The peptide, spin-labeled with methanethiosulfonate nitroxide spin label (MTSL) at position L46C, was reconstituted either into DOPC/POPS or DLPC/DLPS lipid membranes, both prepared in 85:15 molar ratio of non-charged to charged lipid. This work is a continuation of our previous DEER study of this system where we found that the M2TMD_21−49_ tetrameric channel assembles via a dimer intermediate (Georgieva et al., 2015). In the present work, we focused on the effect of amantadine (hydrochloride) on M2TMD_21−49_ conformations for peptide-to-lipid molar ratios (P/L) ranging from 1:18,800 to 1:160. In the presence of amantadine we observe conspicuous changes in the recorded DEER signals in DOPC/POPS membranes for all P/L's. The binding of the drug shifts the M2TMD_21−49_ oligomer equilibrium toward greater content of tetramers, as concluded based on the substantial increase of the DEER signal modulation depth, which increases with oligomeric number (Milov et al., 1984b; Bode et al., 2007; Borbat and Freed, 2007; Jeschke et al., 2009; Bhatnagar et al., 2012; Georgieva et al., 2013, 2015). Consequently, the tetramers must be stronger bound than in the absence of amantadine. Moreover, in the presence of amantadine, the inter-spin distances measured by DEER are shorter by some 5–8 Å compared to the case without amantadine, all other conditions being equal. Thus, binding of amantadine induces closure of the M2TMD C-terminal pore, thereby producing inactive M2 channels. Notably, as opposed to the wild type M2TMD_21−49_, in the case of peptide carrying the G34A drug-resistant mutation (Nguyen and Le, 2015) we did not observe any significant amantadine-induced structural rearrangements at the C-terminus. This indicates that just the binding of amantadine is insufficient for blocking proton conductivity of the M2 proton channel, but a large helix rigid-body movement or bending may be required to squeeze the bundle, thereby closing the proton exit gate. Furthermore, we found that in the membranes of DLPC/DLPS, which are thinner than those of DOPC/POPS, the assembly of M2TMD_21−49_ oligomers was much less efficient, and the effect of amantadine was almost nonexistent. This emphasizes the importance of lipid bilayer thickness and the tuning of its properties in the regulation of the assembly and functioning of M2 protein.

In addition, we applied electron microscopy (EM) to M2TMD_21−49_ peptide, labeled with a gold nanoparticle reagent (Nanogold®) at the same L46C residue and reconstituted into DOPC/POPS bilayers at P/L of 1:10,000. Although we do not quantitatively characterize the oligomeric fractions in this very dilute system, the EM micrographs obtained clearly establish the presence of M2TMD_21−49_ monomers, dimers, and tetramers both in the presence and absence of amantadine, thereby confirming the model based on a strongly-bound dimer intermediate as determined by our previous DEER study on the M2-channel assembly pathway (Georgieva et al., 2015).

## Materials and methods

### Influenza A M2 peptide synthesis and spin-labeling

Wild type (wt) and G34A mutant of M2TMD_21−49_ peptides (with the sequences DSSDPLVVAASIIG[A]ILHLILWILDR**C**FFK, L46C), synthesized commercially (GenScript Inc., USA) to the purity higher than 98%, were received as powders. All procedures for solubilizing and spin-labeling to the efficiency well above 90% (perhaps as high as 98%) were performed as described previously (Georgieva et al., 2015). However, the final buffer contained 50 mM MES (2-(N-morpholino)ethanesulfonic acid) as compared to 25 mM used in the earlier work (Georgieva et al., 2015). Consequently, the final buffer composition was 1 mM β-DDM (n-Dodecyl β-D-maltoside), 50 mM MES pH 5.5, 150 mM NaCl. The comparison of the DEER data for samples with 25 mM and 50 mM MES showed they were nearly identical. The M2TMD_21−49_ peptides were spin-labeled with S-(2,2,5,5-tetramethyl-2,5-dihydro-1H-pyrrol-3-yl)methyl-methanesulfonothioate spin label (MTSL, Toronto Research Chemicals). Spin-labeled peptide concentration after free spin-label removal was 100 μM prior reconstitution into lipid membranes.

### Preparation of lipid membranes and M2TMD_21−49_ peptide reconstitution

All lipids were purchased from Avanti Polar Lipids. Procedures were carried out as described in Georgieva et al. (2015). Chloroform solutions of DOPC/POPS (1,2-dioleoyl-*sn*-glycero-3-phosphocholine:1-palmitoyl-2-oleoyl-*sn*-glycero-3-phospho-L-serine) (di-18:1 PC/16:0/18:1 PS) and DLPC/DLPS (1,2-dilauroyl-*sn*-glycero-3-phosphocholine:1,2-dilauroyl-*sn*-glycero-3-phospho-L-serine, di-12:0 PC/di-12:0 PS) at 85:15 molar ratio of uncharged-to-charged lipid were mixed, dried under vacuum for at least 6 h, and then dissolved in 50 mM MES, pH 5.5, 150 mM NaCl to the final total lipid concentration of 30 mM. Spin-labeled wt and G34A peptides were then added to the lipids. The samples of wt peptide were prepared in several peptide-to-lipid molar ratios (P/L). The ratios were 1:18,800, 1:9400, 1:4100, 1:2300, 1:1650, 1:1300, 1:820, 1:500, 1:235, and 1:160 for DOPC/POPS; and 1:1300, 1:500, 1:160 for DLPC/DLPS. In addition three samples of spin-labeled G34A peptide were prepared in DOPC/POPS for P/L's of 1:1300, 1:500, and 1:160.

### Reacting M2TMD_21−49_ with amantadine

Stock solution of 100 mM amantadine hydrochloride (amantadine) from Sigma-Aldrich was prepared in 50 mM MES, 150 mM NaCl, and subsequently added to samples of wt and G34A mutant of M2TMD_21−49_ for all P/L's and lipid compositions studied to a final concentration of 2 mM, i.e., ca. 5 mol% drug-to-lipid ratio, similarly to previous studies (Thomaston et al., 2013). To ensure uniform distribution of amantadine, the samples were extensively vortexed, frozen in liquid N_2_ and thawed to room temperature (RT) twice. After the last thaw, the samples were kept for 2 min at RT before flash-freezing them in liquid N_2_ for DEER measurements. The whole procedure for reacting M2TMD_21−49_ samples with amantadine took about 4 min, well within the requirements for efficient binding (Salom et al., 2000). Additionally, samples with P/L's of 1:4100 and 1:2300 were incubated with amantadine at RT for 2 h after the last thaw to test the effect of longer reaction. Their respective DEER signals had no detectable dependence on the incubation time (Supplementary Figure 1).

### Preparation of samples for DEER and cw ESR measurements

The following types of samples were used for DEER measurements: (i) DOPC/POPS-reconstituted wt M2TMD_21−49_ in the presence of 2 mM amantadine for all P/L's ranging from 1;18,800 to 1:160; (ii) Amantadine-free wt M2TMD_21−49_ for P/L's of 1:9400, 1:4100, 1:1200, 1:1300, 1:500, and 1:160 in DOPC/POPS membranes; (iii) G34A M2TMD_21−49_ in DOPC/POPS with and without 2 mM amantadineand P/L's 1:1300, 1:500, and 1:160; (iv) wt M2TMD_21−49_ in DLPC/DLPS membranes at P/L's 1:1300, 1:500, and 1:160 without and with amantadine. All samples for DEER measurements had 20% (w/v) glycerol as a cryoprotectant (Georgieva et al., 2015). Sample size was 20 μL in a 2.5 mm O.D. Pyrex tube. The samples were flash-frozen and stored in liquid nitrogen for measurements.

The following samples were used for cw ESR measurements: (i) wt M2TMD_21−49_ in DOPC/POPS with and without amantadine at P/L's of 1:4100, 1:1300, 1:500, and 1:160; and (ii) magnetically diluted at 1:6 molar ratio of spin labeled to unlabeled wt M2TMD_21−49_ in DOPC/POPS, either with or without amantadine, at P/L of 1:160. All samples for cw ESR measurements had no glycerol. All samples for DEER and majority of samples for cw ESR were prepared and measured in duplicates.

### DEER measurements and inter-spin distance reconstruction

DEER measurements were carried out at 60 K using a home-built pulse ESR spectrometer operated at 17.3 GHz in Ku band (Borbat et al., 1997). The standard four-pulse DEER sequence (Pannier et al., 2011) used for detection π/2-π-π pulse sequence with respective pulse widths of 16, 32, 32 ns; a 32 ns π-pulse was used for pumping. This is our preferred setup for the case of more than two coupled spins (Georgieva et al., 2013). The separation between the detection and pump pulse frequencies was 70 MHz. The detection pulses were applied at the low-field edge of the nitroxide spectrum. Typical dipolar evolutions times were in 1.4–2 μs range, with signal averaging time being 2–4 h in most cases, except for the smallest P/L's (1:4100 to 1:18,800), where it was increased to 12–24 h The signal background of the raw DEER data was removed in the standard way (Georgieva et al., 2015) by approximating the latter part of the logarithm of the signal by a second degree polynomial and subtracting it out, followed by proper normalization and origin shift (Supplementary Figures 1, 2). This procedure of background subtraction produced maximum ±2.5% error in the modulation depth for the smallest P/L's, as estimated previously (Georgieva et al., 2015). Taking into account all typical experimental uncertainties, such as introduced in the course of sample preparation and due to, finite reproducibility of resonator tuning and DEER experimental parameters, we accepted more conservative error margins of ±5%. Inter-spin distances were reconstructed from the DEER data, background corrected and normalized as described above and in our previous work (Georgieva et al., 2015), by using the L-curve Tikhonov regularization method (Chiang et al., 2005a) refined when necessary by the maximum entropy method (Chiang et al., 2005b). It should be noted that distances distributions are plotted up to the upper range of 8 nm, which is beyond ~6 nm imposed by the length of data record (~2 μs, except the cases of the largest L/P). This mostly serves to show that baseline fit is good enough by not producing large spurious peaks beyond 6 nm. In some cases, the reconstructed distance distributions were modeled by fitting them to a sum of two Gaussians using a non-linear least-square (NLLS) curve fitting algorithm as implemented in the OriginLab 8.0 software package (OriginLab, Inc.).

### cw ESR measurements

The cw ESR measurements were performed at X-band (microwave frequency ca. 9.4 GHz) on liquid samples at room temperature (ca. 25°C), and also at controlled temperatures of 21°C and 40°C, as well as on frozen samples at −100°C (Supplementary Figures 3, 4). The cw ESR spectra were recorded, using the Bruker ELEXIS E500 (Bruker, Billerica, USA) ESR spectrometer equipped with ER 4122-SHQE super-high Q resonator and ER4131VTVT-31 variable temperature accessory. The microwave power and modulation amplitude were 1.26 mW and 2.2 G for the measurements on liquid samples and 50 μW and 2 G for frozen samples. For estimation of spin-label dynamic parameters the cw ESR spectra for samples of wt M2TMD_21−49_ in DOPC/POPS membrane for P/L's of 1:1300 and 1:500, both with and without amantadine, were fitted using the NLLS program (Budil et al., 1996) and the MOMD model.

### Concentration profile analysis

This analysis was performed using the NLLS procedure based on Levenberg-Marquardt algorithm implemented in MATLAB® (Georgieva et al., 2015).

### Attaching gold nanoparticle tag to wt M2TMD_21−49_ peptide

The detergent-reconstituted peptide was mixed with monomaleimido functionalized gold nanoparticles (1.4 nm Monomaleimido Nanogold® from Nanoprobes) at 1:1 molar ratio in the buffer of 100 mM sodium phosphate pH 6.0, 5 mM EDTA and 1 mM DDM, and incubated at RT for 6 h. When attached, Nanogold particles add ca. 15 kDa to the molecular weight of the monomeric peptide, thus producing a labeled peptide of average molecular weight >18 kDa. Hence, a tetramer with all subunits labeled should have a molecular weight exceeding 60 kDa. To remove the unreacted Nanogold, the sample was washed at least five times with the buffer of 25 mM MES pH 5.5, 150 mM NaCl and 1 mM DDM using 30 kDa MWCO concentrator and keeping the peptide concentration in the range where the tetramer is dominant, i.e., above peptide to detergent molar ratio (P/D) of 1:40 (Georgieva et al., 2015). Then the peptide sample was diluted with the buffer to a P/D, which was higher than 1:3500 where peptide is mostly monomeric and dimeric and concentrated using 10 kDa MWCO filter to remove the monomeric unlabeled peptides. This procedure was repeated five times. Thus, we are confident that the final sample was highly enriched in Nanogold labeled M2TMD_21−49_ peptide. The final peptide concentration was estimated from the Nanogold particle extinction coefficient of 156,000 M^−1^cm^−1^ at 420 nm (as specified by the manufacturer).

Thereafter, the labeled peptide was mixed with Triton-100 destabilized DOPC/POPS (85:15 molar ratio) lipid suspensions in the buffer of 25 mM MES pH 5.5 and 150 mM NaCl followed by incubation at RT for 1 h. The P/L was ca. 1:10,000 within the accuracy of peptide concentration determination. Then the sample was dialyzed for 48 h at 4°C. Liposomes were prepared using a mini extruder (Avanti Polar Lipids, Inc.) and 0.4 micron polycarbonate membrane.

### Electron microscopy

Negative stain TEM: 5 μL of sample consisting of lipid vesicles with Nanogold labeled M2TMD_21−49_, prepared either with or without amantadine, were placed onto freshly carbon-coated and plasma treated hexagonal EM grids (300 mesh, Ted Pella, Inc., Redding, CA, USA) and stained with 1% Uranyl acetate (UA). Thereafter, digital micrographs were collected with a JEOL - JEM2100 LaB6 equipped transmission electron microscope (TEM) (JEOL, Ltd) operated at 200 kV and equipped with a 4 × 4 US4000 CCD camera (Gatan) at a low dose conditions (<16 e^−^/Å^2^), and at 2.8 Å/pixel final resolution (× 54,323 final magnification, Supplementary Figure 5).

Cryo-EM: Freezing was done using Lacey 200 mesh EM grids (TedPella, Inc). Plasma clean 15 s, BF = 0, BT = 4 s. Sample volume was 2.5 μL. The excess liquid was blotted and the grids quickly plunged (~2000°C/s) into liquid ethane, cooled down by liquid nitrogen to obtain amorphous ice in a Vitrobot Mark IV (FEI, Millsboro, OR, USA). The grids were transferred and observed at liquid nitrogen temperature. Digital micrographs were recorded on TEM JEOL JEM2200 TEM (JEOL Ltd) operated at 200 kV and equipped with a field emission gun energy filter and 4 × 4 K US4000 CCD camera (Gatan, Inc), at low- dose conditions (15 e^−^/Å^2^) and 2.9 Å/pixel resolution.

## Results

### Binding of amantadine shifts the equilibrium to more stable M2TMD_21−49_ tetramers in DOPC/POPS membranes

**Figure 2:**
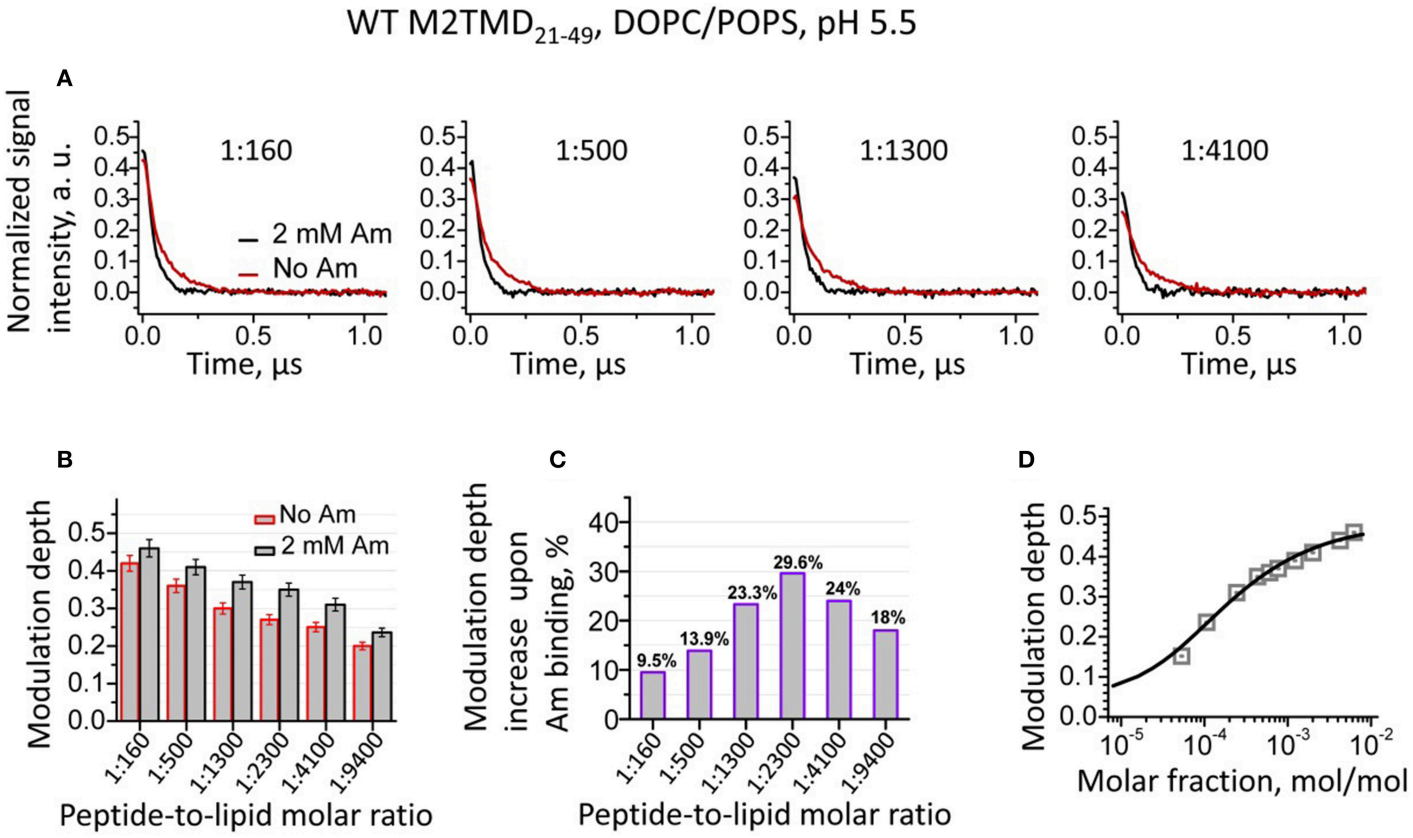
**(A)** Background subtracted and normalized (as detailed in the supplement, cf. Supplementary Figure 2) DEER data for spin-labeled M2TMD_21−49_ in DOPC/POPS membranes at pH 5.5 and four P/L's, namely 1:160, 1:500, 1:1300, and 1:4100. The data without and with (2 mM) amantadine are in red and black, respectively. Samples with amantadine were incubated for 2 min at RT after two cycles of freeze/thaw, (cf. Experimental Procedures). Only the early 1.1 μs of the DEER signals are shown for clarity. **(B)** The DEER modulation depths for samples without and with amantadine for P/L's 1:160, 1:500, 1:1300, 1:2300, 1:4100, and 1:9400. The error bars correspond to the maximum uncertainty of ±5% in the modulation depth estimation (cf. Experimental Procedures). **(C)** Relative increase in DEER modulation depth upon addition of amantadine for the P/L's in **(B)**. **(D)** DEER signal modulation depth concentration profile (open gray squares) is plotted vs. M2TMD_21−49_ molar fraction in DOPC/POPS lipid mixture with amantadine. The solid line is the fit of these data to the equilibrium model based on tandem momomer↔dimer↔tetramer. The obtained equilibrium constants, *k*_2d_, *k*_4d_ are 39·10^−6^MF and 42·10^−6^MF, pointing to stronger bound tetramers than in the absence of the drug.

We studied the effect of drug-binding on the structure of M2TMD_21−49_ in DOPC/POPS by recording the DEER time-domain signals for a series of spin-labeled samples, which were either amantadine-free or contained 2 mM amantadine. Experiments were conducted over a broad range of P/L's from 1:18,800 up to 1:160. We observed that for the same P/L binding of amantadine led to an increase in the DEER modulation depth Δ (the DEER signal amplitude at zero time in our plots) compared to the amantadine-free sample (Figures 2A,B). This effect was detected for all P/L's studied; although to a different extent (Figures 2A–C), with a maximum increase of 23–30% at P/L's between 1:1300 and 1:4100 (Figure 2C).

Here we remind the reader that DEER modulation depth is defined as Δ(*p*) = 1 − DEER(t = ∞)/DEER(t = 0) and directly depends on the number of interacting spins as Δ (*p, N*) = [1 − (1 − *p*)^*N*−1^], where *p* is the fraction of spins flipped by the pump π-pulse and *N* is the number of interacting spins (Milov et al., 1984b; Bode et al., 2007; Jeschke et al., 2009; Bhatnagar et al., 2012; Borbat and Freed, 2014). We used this spin-counting capacity of DEER spectroscopy to elucidate the mechanism of assembly of M2TMD_21−49_ in our previous work(Georgieva et al., 2015).

As we previously established, within the broad range of P/L's studied, the drug-free M2TMD_21−49_ exists as an admixture of monomers, dimers, and tetramers, with the population of each of these oligomeric forms being strongly dependent on P/L (Georgieva et al., 2015). In this work, the increase in the DEER modulation depth shows that binding of amantadine dramatically shifts the equilibrium of M2TMD_21−49_ oligomeric species toward the higher order oligomers, enriching tetramers. We have no reason to believe that other intermediate oligomers, i.e., trimers, are formed, since their existence was not detected by any technique previously employed. Presence of very short distance in amantadine-free or amantadine-bound conformations of M2TMD_21−49_, which would reduce the modulation depth, could also be ruled out, since no such distances were detected by low temperature cw ESR (Georgieva et al., 2015; Supplementary Figure 4). To confirm our hypothesis, we fitted the experimental DEER modulation depth concentration profiles (Supplementary Figure 6), for the samples with amantadine to a two-stage equilibrium that is $\text{monomer}\overset{k_{2\text{d}}}{↔}\text{dimer}\overset{k_{4\text{d}}}{↔}\text{tetramer},$ as previously described (Georgieva et al., 2015). There was good agreement between the model and the experiment data (Figure 2D). Thus, in the presence of amantadine, we obtained dissociation constants *k*_2d_ (expressed as mole fraction, MF) of 39 ppm and *k*_4d_ of 42 ppm. For comparison, these constants for drug-free M2TMD_21−49_ were 15·ppm (P/L ~ 1:65,000) and 448·ppm (P/L of 1:2230), as previously determined (Georgieva et al., 2015). The 10-fold reduction of *k*_4d_ in the presence of amantadine and the close values of *k*_2d_ and *k*_4d_ shows that binding of amantadine strongly stabilizes M2TMD_21−49_ tetramer, whereas the values of *k*_2d_ remain reasonably close in both cases. The closeness of the two dissociation constants in the case of amantadine results in a greater uncertainty in the *k*_2d_ value determination, so it would be premature to construe that amantadine has any effect or even binds to dimers. Our observations are in the agreement with the previously reported stabilizing effect of amantadine on M2TMD tetramer in dodecylphosphocholine micelles at both high and low pH (Salom et al., 2000).

**Figure 3:**
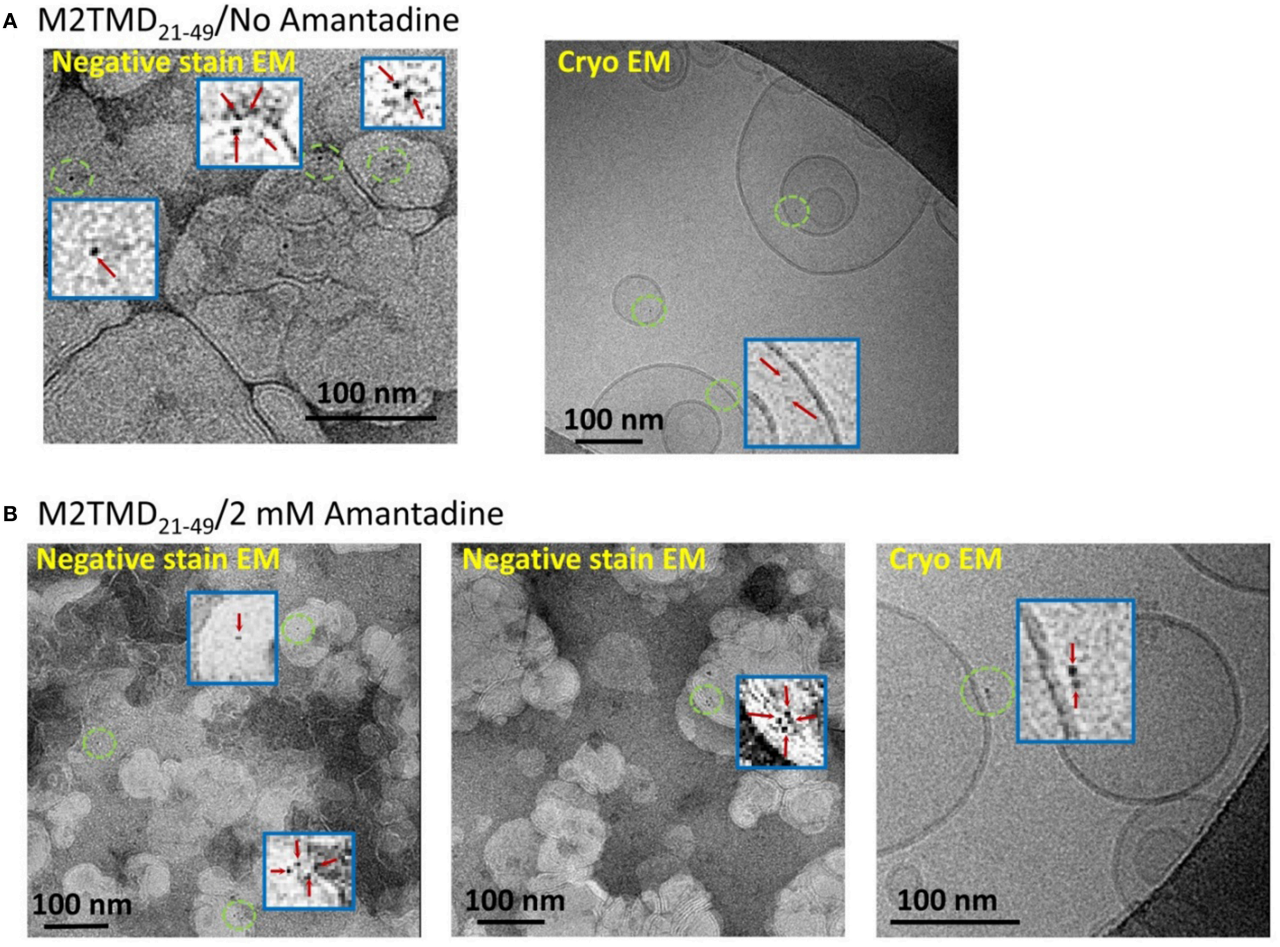
**EM micrographs of M2TMD_21−49_ nanogold labeled at position L46C and reconstituted into DOPC/POPS membranes**. The data shown in **(A,B)** are those without and with 2 mM amantadine, respectively. In both cases the P/L was 1:10,000. The blue boxes are the areas contained inside green circles magnified by a factor of 20 to emphasize monomers, dimers and tetramers of M2TMD_21−49_.

To further support our DEER results, we conducted electron microscopy (EM) experiments on M2TMD_21−49_ fully-labeled at the same L46C residue with monomaleimido-gold nanoparticles (Nanogold®). Two methods were used—negative staining TEM and cryo EM. The obtained data for sample of M2TMD_21−49_ in DOPC/POPS bilayers at a P/L of ~1:10,000 with and without amantadine are shown in Figures 3A,B, respectively. In the EM micrographs one can distinguish monomers, dimers, and tetramers of nanogold-labeled M2TMD_21−49_, albeit obtaining quantitative information about the fraction of these oligomeric species at such a high dilution was outside the range of our EM experiments. Given the careful iterative procedure for the removal of unlabeled peptide and unreacted nanogold particles (Experimental Procedures), we are confident that no significant amount of unlabeled M2TMD_21−49_ was present in the samples, therefore the type of gold nanoparticle clusters, discernable in EM micrographs, faithfully reports on the type of M2TMD_21−49_ oligomeric species present under the conditions of our experiment.

### In DOPC/POPS membranes the C-terminal pore of M2TMD closes upon binding of amantadine

**Figure 4:**
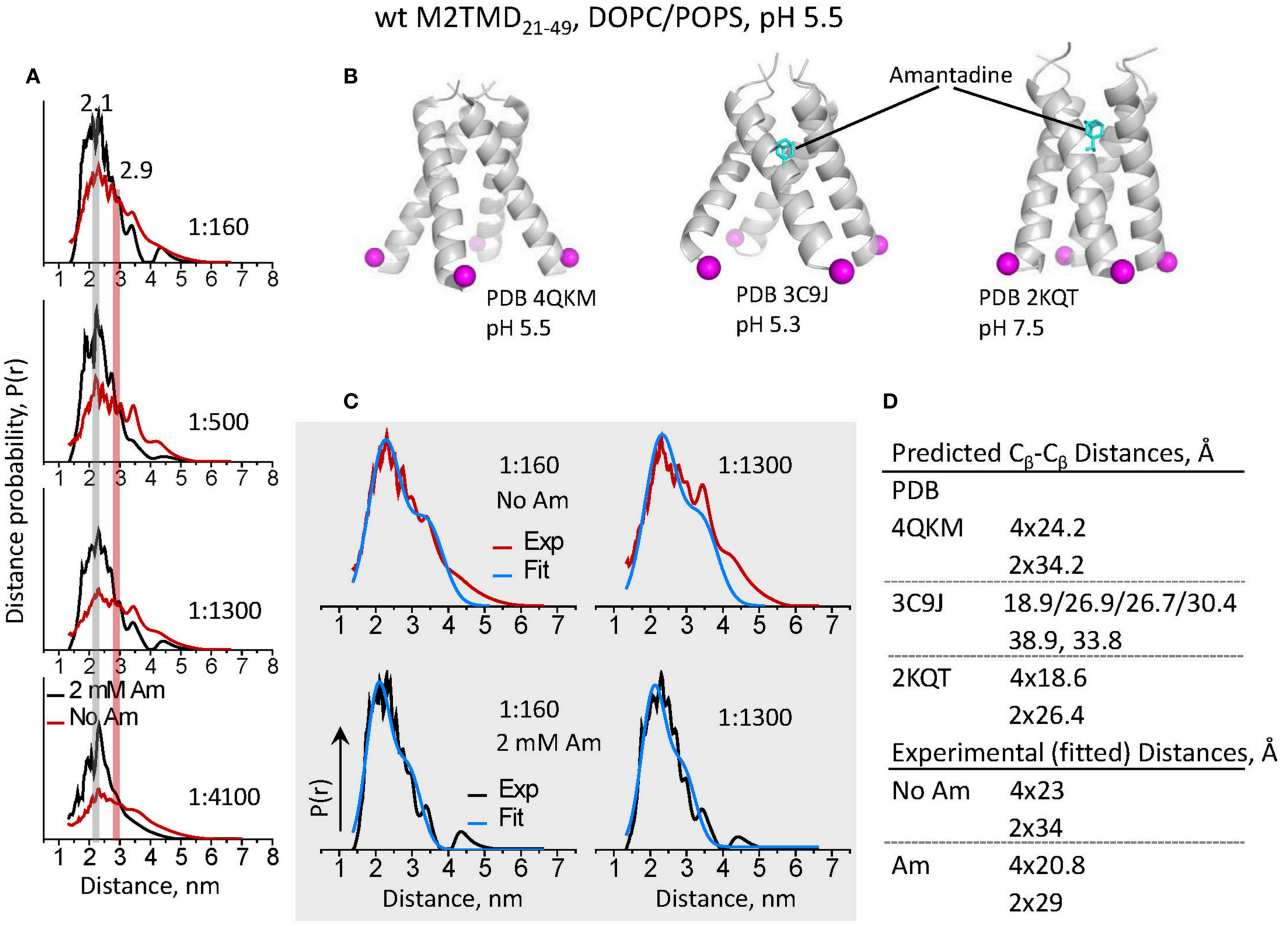
**DEER distances reveal a large conformational motion of M2TMD_21−49_ channel leading to the closure of C-terminal pore triggered by amantadine binding. (A)** Inter-spin distances for spin-labeled L46C reconstructed from the DEER time-domain signals shown in Figure 2 for P/L's 1:160, 1:500, 1:1300, and 1:4100. Data for M2TMD_21−49_ samples without and with 2 mM amantadine are plotted in red and black, respectively. A shift by 5–8 Å to shorter distances was observed in all cases upon binding of amantadine. **(B)** Positions of residues L46 C_β_-atomsin M2TMD tetramer based on published amantadine-bound structures at pH 5.5 in lipid cubic phase (X-ray); pH 5.3 in detergent (X-ray); and in lipid at pH 7.5 (ssNMR). Contrary to our observations, in the amantadine-bound low-pH crystal structure, the C-terminal of M2TMD tetramer is widely open and asymmetric. **(C)** The experimental distances for P/L's 1:160 and 1:1300 are shown along with the two-Gaussian fit (in blue). **(D)** A compendium of C_β_-C_β_ distances based on the available atomic-resolution structures (shown in **C**) and those extracted from the two-Gaussian fitting of DEER distances.

In addition to increased modulation depth of the recorded DEER signals for the samples with amantadine, we observed faster decaying DEER signals (Figure 2A) in spin-labeled wt M2TMD_21−49_. These signals correspond to inter-spin distances shorter by some 5–8 Å than those in the absence of the drug, all other conditions being equal (Figure 4A). Furthermore, the distance distributions for amantadine-bound M2TMD_21−49_ were significantly narrower than those for the drug-free peptide (Figure 4A). To further address how these structural transitions may be coupled with channel inhibition, we analyzed in more detail our experimental inter-spin distances distributions for samples without and with amantadine and within the context of existing structural data. Specifically, we compared the distances from our experiment to those derived based on the available structures. As this manuscript was being finalized, the structure of amantadine-free M2TMD at pH 5.5 has emerged, solved by X-ray crystallography in the lipid cubic phase (Thomaston et al., 2015). We thus employed this structure as a template to rationalize our DEER distance distributions by fitting them to a weighted sum of two Gaussians in a ratio of 4:2 (proximal-to-lateral distances). In the fittings, the initial guesses for Gaussian means corresponded to the C_β_-C_β_ distances in the crystal structure, which are 24.2 Å (proximal) and 34.2 Å (lateral) in the four-fold symmetric tetramer (PDB code 4QKM, cf. Figure 4B). Certainly this model represented the experimental distance distributions well, and from the fittings we obtained *r*_1_ = 23 Å with σ_1_ = 9 Å, and *r*_2_ = 34 Å with σ_2_ = 9 Å, where *r* and σ are the Gaussians mean and standard deviation. These *r*_1_ and *r*_2_ values agree well with the expected *r*_lateral_ to *r*_proximal_ 2^1∕2^ ratio in a homotetramer with the four-fold symmetry, as discussed previously (Dalmas et al., 2012). These fittings along with the experimental distance distributions for P/L's of 1:160 and 1:1300 are shown in Figure 4C. Notably, the same set of parameters used in the fittings for P/L of 1:1300 did not account for noticeable content of longer range experimental distances, which we previously attributed to M2TMD_21−49_ dimers (Georgieva et al., 2015). Thus, the tetramer produced shorter distances, both proximal and lateral, than the dimer, which most likely is due to a more compact nature of the four-helix bundle.

Next, we aimed to characterize the conformation of the C-terminal of M2TMD channel when bound to the amantadine channel blocker. To do so, we first used the crystal structure of M2TMD tetramer with amantadine bound to the central pore at pH 5.3 (PDB ID 3C9J) (Stouffer et al., 2008). However, our experimental distances were significantly shorter and the distributions were considerably narrower than those in the crystal structure (Figure 4). Obviously, in this structure the helices splay apart at the C-terminal forming a wide open pore, considerably wider than in the structures observed at elevated pH (Schnell and Chou, 2008; Andreas et al., 2010; Sharma et al., 2010). Also, the arrangement of helices, including helix rotation, provides a degree of asymmetry in the C-terminal with four distinct proximal distances of 18.9 Å, 26.9 Å, 26.7Å and 30.4 Å, and two lateral distances of 38.9 Å and 33.8 Å between the L46 C_β_ atoms in M2TMD (Figure 4B). This apparent disagreement between the DEER-derived and crystallography determined distances could be a result of lipid bilayer effects on the M2TMD structure (see below), but could also be because of the G34A mutation in the crystalized construct (Stouffer et al., 2008).

To address this discrepancy, we tested how the distances from highly symmetric and much more closed ssNMR structure of amantadine-bound M2TMD channel in DMPC membranes at pH 7.5 (Cady et al., 2010) match our results (Figure 4B). Again, we fit the DEER distances for spin-labeled residue L46C to a sum of two Gaussians with contributions 4:2, and obtained *r*_1_ = 20.8 Å with σ_1_ = 6.7 Å, and *r*_2_ = 29 Å with σ_2_ = 7 Å, which are very close to the C_β_-C_β_ distances, i.e., 18.6 Å and 26.4 Å, in the NMR structure, and agree well with a highly symmetric homotetramer. In this case the Gaussian fittings better represented the majority of experimental distances even at P/L of 1:1300, as expected from the increase in the population of M2TMD_21−49_ tetramers. Furthermore, the Gaussian widths (σ), extracted from the fittings, i.e., representing the widths of the distance distributions, were narrower by ca. 2 Å in the presence of amantadine than those without drug, and the ratio between the mean proximal and lateral distance was closer to that of a tetramer with a four-fold symmetry. This indicates that amantadine-stabilized structure is less heterogeneous and more symmetric.

**Figure 5:**
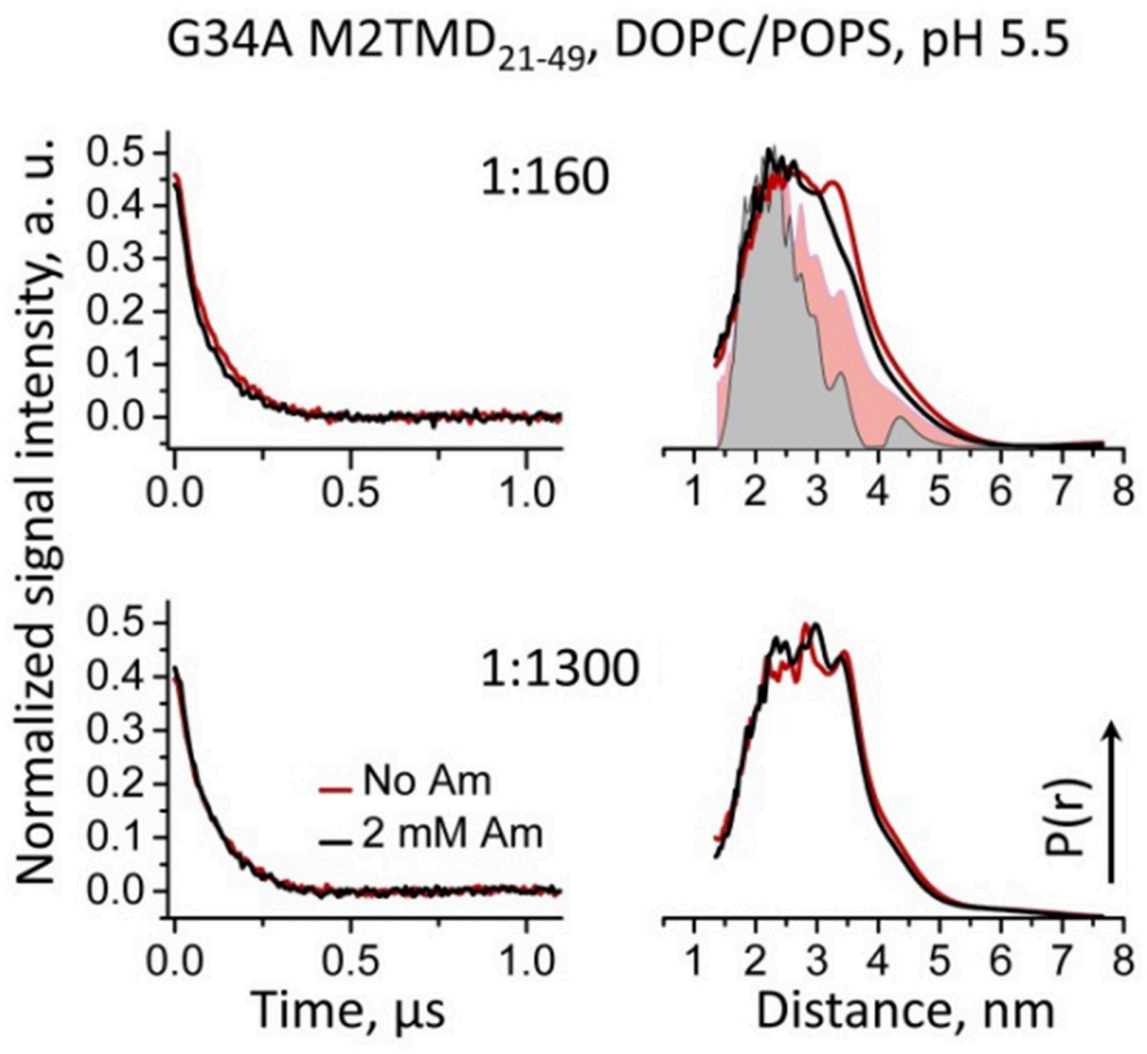
**Background-subtracted normalized time-domain DEER data (left) and reconstructed distance distributions (right) for spin-labeled L46C residue in G34A mutant of M2TMD_21−49_ reconstituted in DOPC/POPS membranes are shown for P/L's of 1:160 and 1:1300**. Only the early 1.1 μs of the DEER signals are shown for clarity. The data with and without amantadine are solid lines drawn in black and red, respectively. Inter-spin distances do not change upon the addition of amantadine, and they are in the range of 20 to 40 Å. For comparison, the distance distributions for wt M2TMD_21−49_ are shown in the upper right panel as pink and gray flooded areas. In this case the inward movement is pronounced.

To further establish the reason for the discrepancy between the crystal structure and our results, we measured DEER distances between L46C residues in M2TMD_21−49_ carrying the mutation G34A. Indeed these distances were distributed between 20 and 40 Å, which is a significantly broader range than in wt M2TMD_21−49_ and is closer to the predictions based on MTSL rotamer libraries (Polyhach et al., 2011) generated for low-pH crystal structure (Stouffer et al., 2008) (Figure 5). Strikingly, binding of amantadine had marginal if any effect on M2TMD C-terminal conformation, manifested by almost unchanged time-domain DEER signals and correspondent inter-spin distances. This result points to a potentially critical functional role of the G34 residue, including drug resistance. Furthermore, the G34A mutation itself did not reduce the M2TMD_21−49_ self-association, based on the DEER modulation depth. In fact, the DEER modulation depths for P/L's 1:1300 and 1:160 were even slightly greater than those for wt peptide under equal conditions (Figures 3A, 5), but answering the question whether this mutation has any tetramer stabilizing effect is beyond the scope of this study.

### The hydrophobic thickness of lipid bilayer influences the structure of M2TMD_21−49_ and its interaction with amantadine

**Figure 6:**
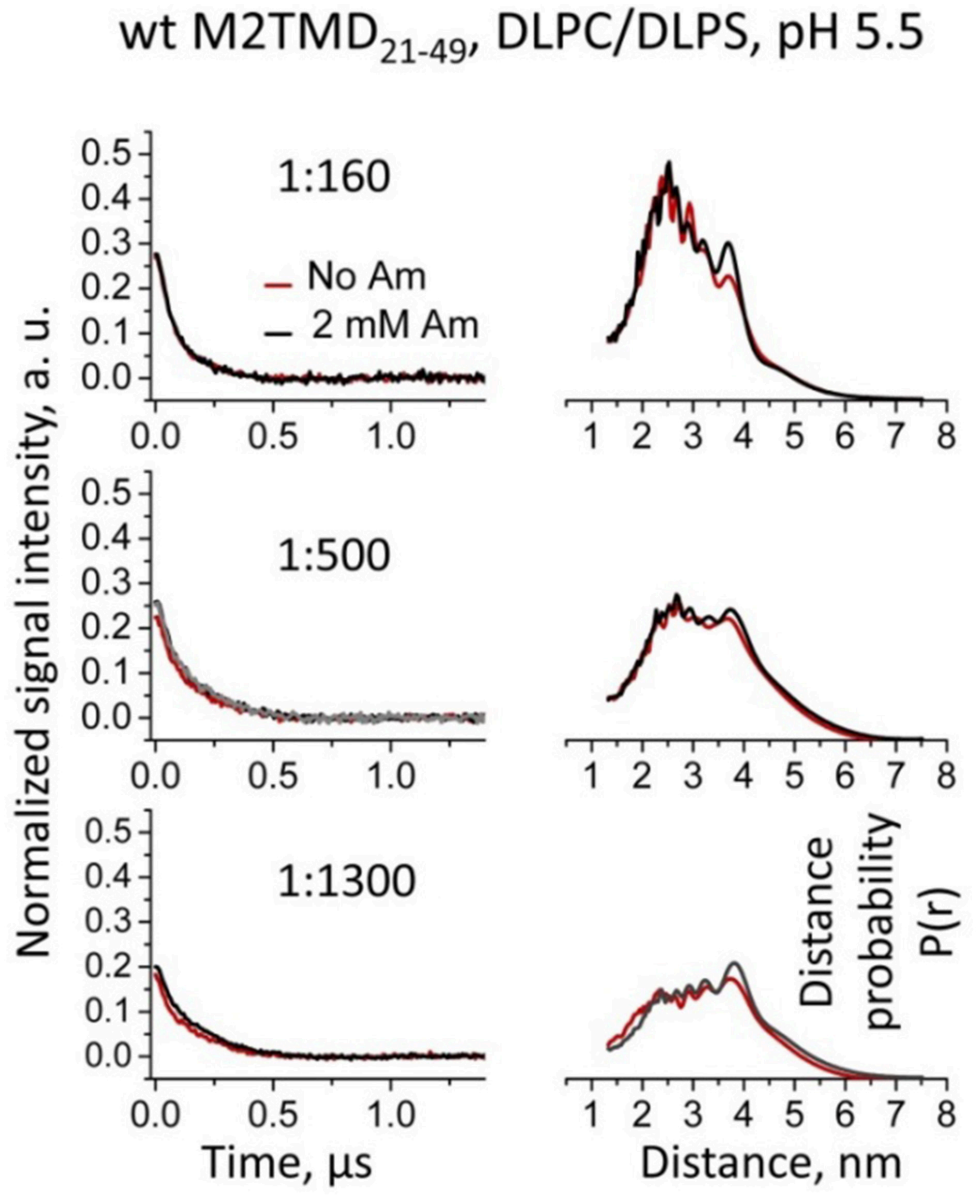
**Effect of amantadine on DEER data is almost negligible for WT M2TMD_21−49_ residing in DLPC/DLPS membranes**. Background subtracted and normalized DEER time-domain data (left) and corresponding distances (right) for spin-labeled residue L46C at P/Ls of 1:160, 1:500, and 1:1300 are shown. Only the early 1.1 μs of the DEER signals are shown for clarity. No detectable or very small changes in the DEER signals and corresponding distances were observed upon addition of amantadine. Longer incubation with amantadine (1 h at RT) did not produce any additional effect on the DEER data (gray signal in the middle left panel for P/L of 1:500) compared to standard incubation of ~4 min.

Recognizing the role of the lipid bilayer hydrophobic thickness on the structure and inhibition of M2TMD_21−49_ was an extra goal of the current study. We reported that self-association of M2TMD_21−49_ is more efficient in membranes with hydrophobic thickness matching the 28.5 Å length of the M2TMD transmembrane helix, such as DOPC/POPS, compared to thinner membranes of DLPC/DLPS (Georgieva et al., 2014). Accordingly, we studied the effect of amantadine on M2TMD_21−49_ reconstituted in DLPC/DLPS at 85:15 mol% vs. DOPC/POPS taken at the same ratio. Interestingly, in the thinner membranes the effect of amantadine on the shape of DEER signals for the same P/L's, particularly on their modulation depth, was almost negligible (Figure 6, left). Moreover, in DLPC/DLPS the shapes of DEER signals were significantly different from those in DOPC/POPS, leading to longer reconstructed inter-spin distances for spin-labeled residue L46C (Figure 6, right). This suggests that M2TMD helix may adjust to the hydrophobic mismatch by increasing its tilt angle in agreement with similar finding by NMR (Hu et al., 2011). Finally, in DLPC/DLPS the DEER distances for P/L's of 1:1300, 1:500, and 1:160 do not change upon the addition of 2 mM amantadine. Consequently, amantadine-induced tetramer stabilization and closure of C-terminal proton exit pore does not occur in this particular membrane environment or these effects are rather small to be conclusive.

## Discussion

In the current study, we aimed to characterize in greater detail the conformational transition from active to inhibited M2TMD channel and the oligomeric profile under low pH conditions (pH 5.5), which is close to those found in endosomes, where the M2 proton channel is conductive. Understanding in detail how the inhibition occurs is desirable for designing new and more efficient anti-influenza drugs. Yet, our present knowledge is insufficient. Therefore, we studied the M2TMD_21−49_ construct reconstituted in DOPC/POPS and DLPC/DLPS membranes, with or without M2 channel blocker amantadine, as well as for the case of the G34A mutation.

In our previous work we found that the transmembrane domain of influenza A M2 protein assembles into a tetramer via a tight dimer intermediate (Georgieva et al., 2015). Here, the results from DEER spectroscopy (Figure 2) and EM (Figure 3) show that just as for the drug-free channel, in the presence of amantadine M2TMD_21−49_ there is also a mixture of monomers, dimers, and tetramers when residing in bilayers of DOPC/POPS, whose hydrophobic thickness closely matches the length of the M2 transmembrane helix (Kučerka et al., 2005; Georgieva et al., 2015). Indeed, our observations agree with a dimeric intermediate, which was previously predicted by MD simulations of the M2TMD assembly process (Carpenter et al., 2008).

Furthermore, our results strongly suggest that the inhibition of the M2TMD proton channel occurs via drug-induced tetramer stabilization and C-terminal channel closure. The detected increase in the DEER modulation depth in the presence of amantadine vs. no amantadine (Figure 2) within a broad range of P/L's from 1:18,800 to 1:160 shows that the binding of drug shifts the equilibrium toward a greater content of tetramers in agreement with previous observations for M2TMD in dodecylphosphocholine micelles (Salom et al., 2000). Using the detailed DEER-based analysis of oligomer equilibria in M2TMD assembly pathway (Georgieva et al., 2015), we estimated the monomer↔dimer and dimer↔ tetramer dissociation constants *k*_d2_ and *k*_d4_ of 39.10^−6^ and 42.10^−6^ MF, respectively, in the presence of the drug. The very close values of these constants points toward a much stronger amantadine-bound tetrameric species, compared to the amantadine-free system for which we previously reported *k*_d2_ and *k*_d4_ of 15·10^−6^ MF (P/L~1:65,000) and 448·10^−6^ MF (P/L of 1:2230) respectively (Georgieva et al., 2015). The M2TMD tetramer dissociation at acidic pH, observed by us (Georgieva et al., 2015) and others (Salom et al., 2000), was ascribed to increased protonation of the H37 tetrad and electrostatic repulsion (Salom et al., 2000). On the contrary, when amantadine binds to M2TMD its charged ammonium group forms a hydrogen bond with the neutral imidazole side chain of the histidine residue, thus expunging the proton and reducing the protonation state of the system (Gandhi et al., 1999; Salom et al., 2000; Hu et al., 2007) resulting in a more stable tetramer.

In addition to M2TMD_21−49_ tetramer stabilization, the observed shift at the C-terminus toward shorter distances by 5–8 Å with the addition of amantadine reveals that in DOPC/POPS membranes M2TMD_21−49_ tetramer undergoes large scale conformational rearrangements leading to C-terminal pore closure. To the best of our knowledge, this is the first observation of such a large conformational change from active to inhibited forms of M2TMD at conditions of low pH (pH 5.5). Moreover, our experiments on M2TMD_21−49_ G34A mutant strongly suggest the critical role of this glycine residue in M2 channel inhibition. This is also supported by the presence of G34A mutation in some amantadine resistant influenza strains (Nguyen and Le, 2015). That is, the ability of M2TMD tetramer to perform proton exit gate closure is inhibited by substituting the G34 residue for alanine and this could be the main reason for the drug-resistance of this mutant. Since the drug-bound M2TMD construct carrying this mutation was previously crystallized (Stouffer et al., 2008), we assume that the observed effect is not a result of abolished amantadine binding. It is likely that the G34 residue serves as a “gating hinge,” similar to ligand- and voltage-gated K^+^ channels (Magidovich and Yifrach, 2004; Chakrapani and Perozo, 2007; Hulse et al., 2014). It should be noted that a kink and a varying extent of conformational heterogeneity at this position in the M2 TM helix was observed by NMR for M2 channel for a range of conditions including the binding of drug at high pH (Hu et al., 2006, 2011; Cady et al., 2010), and it was also predicted from MD simulations (Yi et al., 2009). However, altering the drug-binding site such that it prevents the interaction of amantadine with H37 to change the total protonation state of the histidine tetrad could also be possible.

The detailed DEER-derived distance analysis by fitting to two-Gaussians (Figure 4C, upper panel) suggests that non-inhibited M2TMD_21−49_ tetramer is conformationally more heterogeneous than the amantadine-bound one, and possibly even slightly asymmetric at acidic pH, in agreement with previous observations at higher pH's (Sharma et al., 2010; Andreas et al., 2015), and this could be a prerequisite for channel function. On the contrary, the similar distance analysis in the presence of amantadine shows that the M2TMD_21−49_ tetramers, which are almost fully assembled at P/L's greater than 1:1300, are highly symmetric and more conformationally restricted. This is also supported by the cw EPR measurements taken on the samples of spin-labeled M2TMD_21−49_ with and without the addition of amantadine, from which we found by NLLS spectral fitting that binding of the drug increases the local ordering of the nitroxide spin-label, thereby contributing additional broadening to the MOMD lineshape (Supplementary Figure 3). This indicates that local conformational changes leading to a drug-induced channel confinement restrict the spin-label conformations. All these results taken together suggest that the inhibited form of M2TMD_21−49_ channel is a highly symmetric compact tetramer.

The observed inefficient M2TMD_21−49_ assembly into tetramers in thinner DLPC/DLPS membrane, which is based on the much reduced DEER modulation depth, i.e., Δ < 0.3 at the highest 1:160 P/L used (Figure 6), as well as the lack of effect of amantadine on the inter-spin distance distributions, demonstrates the importance of the match of bilayer hydrophobic thickness on M2TMD self-association and possibly function, as is the case with other membrane proteins (Killian, 1998). It should be noted that in the absence of any data on the binding of amantadine to M2TMD_21−49_ oligomers in DLPC/DLPS bilayers, any reduced affinity to the drug cannot be entirely ruled out. It is expected that protein-drug interaction is dependent on favorable arrangement of helices in M2TMD_21−49_ oligomers. Thus, any unfavorable energetics of M2TMD oligomer insertion into the lipid bilayer could produce penalties for both efficient tetramer formation and drug binding. Indeed, the different degree of assembly of M2TMD_21−49_ in lipid bilayers differing in hydrophobic thickness raises a question about the oligomeric state of M2 as well as about the mechanisms of inhibition on the secretory pathway of this protein when residing in membranes of cellular organelles and cell walls with unequal thickness (Mitra et al., 2004). It would be important in the future to conduct studies clarifying these issues.

Our study, aimed at contributing to the development of M2-based anti-influenza pharmaceuticals, adds to our knowledge of how M2TMD channel conformational states and its environment are related to its function and inhibition. Our spectroscopic data shed light on channel conformations as a function of pH, membrane morphology, the presence of anti-M2 drug, and mutations. Particularly, in the extensively studied topic of the interaction with amantadine, we were able to reveal the drug-induced C-terminus squeezing motion associated with channel closure at pH 5.5, and the absence thereof in the case of G35A mutation. Notably, unambiguous spectroscopic information on the conformational response of M2TMD to binding of anti-M2 drug was provided under pH conditions similar to those in the endosome.

## Author contributions

EG and PB conceived the study and designed experimental approaches. EG and NK carried out the molecular biology experiments, i.e., peptide solubilization, spin-labeling, reconstitution in lipid membranes, and binding of amantadine. EG and PB performed the DEER and cw ESR measurements. EG, PB, and NK analyzed and EG, PB, NK, and JF interpreted the ESR data. ZL simulated the cw ESR spectra. SS and EG designed the EM experiment. EG developed the WT M2TMD_21−49_ labeling with monomaleimido gold nanoparticles. KG and SS conducted the EM experiment and analyzed the data. SS, KG, EG, and PB interpreted EM data. EG, PB, and SS drafted the manuscript. All authors wrote and approved the final version of the manuscript.

## Funding

This work was supported by NIH/NIBIB grant R01EB003150 and NIH/NIGMS grant P41GM103521 to JF, and funds from Sealy Center for Structural Biology and Molecular Biophysics to SS.

### Conflict of interest statement

The authors declare that the research was conducted in the absence of any commercial or financial relationships that could be construed as a potential conflict of interest.
