# Supplementary material for: Conformational Response of Influenza A M2 Transmembrane Domain to Amantadine Drug Binding at Low pH (pH 5.5)
Source: Front Physiol. 2016 Jul 29;7:317. doi: 10.3389/fphys.2016.00317 (PMC4965473; doi:10.3389/fphys.2016.00317)
Supplement: Supplementary file 1 [file Presentation1.pdf]

Supplementary information

Conformational response of influenza A M2 transmembrane domain to amantadine drug binding at low pH (pH 5.5)

Elka R. Georgieva[1,2†*], Peter P. Borbat[1,2], Kirill Grushin[3‡], Svetla Stoilova-McPhie[3], Nichita J. Kulkarni[4], Zhichun Liang[1,2], Jack H. Freed[1,2*]

[1] Department of Chemistry and Chemical Biology and [2] National Biomedical Center for Advanced ESR Technology, Ithaca, NY, USA

[3] Department of Neuroscience and Cell Biology, Sealy Center for Structural Biology and Molecular Biophysics, University of Texas Medical Branch at Galveston, Galveston, TX, USA

[4] College of Art and Sciences, Cornell University, Ithaca, NY, USA

[*]Correspondence

Elka R. Georgieva
erg54@cornell.edu

Jack H. Freed
jhf3@cornell.edu

[†] Present address: Department of Physiology and Biophysics, Weill Cornell Medicine, New York, NY, USA

[‡] Present address: Department of Cell Biology, School of Medicine, Yale University, New Haven, CT, USA

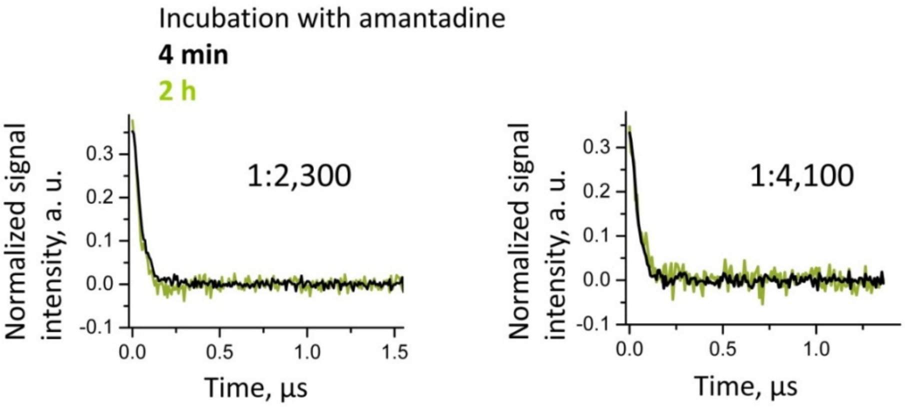

Supplementary Figure 1. DEER signal dependence on incubation time after the addition of amantadine. Background-subtracted and normalized time-domain DEER data for spin-labeled $M2TMD_{21-49}$ in DOPC/POPS at P/L's of 1:2,300 and 1:4,100 incubated with 2 mM amantadine for 4 min (the standard incubation time for this study, cf. Experiment) and 2 h at room temperature (RT). In both cases, after the standard procedure of intermolecular decay and constant background removal (cf. Supplementary Fig. 2 below) no differences in the DEER modulation depth versus incubation time could be noticed.

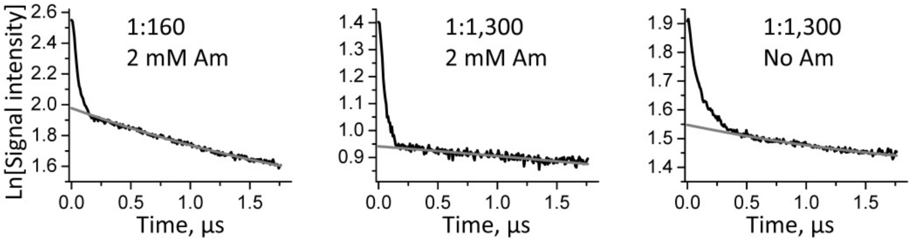

Supplementary Figure 2. Intermolecular decay removal by subtracting out a second-degree polynomial fitted to the asymptotic background of logarithm of the primary DEER signal. After taking the antilog of the remainder, the resulting intramolecular DEER signal, $V(t)$ can be modified as $(V(t) - 1)/V(0)$ to give DEER modulation depth Δ at zero evolution time and 0 asymptotically. This is our preferred way to present DEER data. (Alternatively $V(t)$ may be normalized to unity at zero time to give $1 - \Delta$ asymptotically, cf. Supplementary Fig. 6 below). This procedure of background subtraction produces a small error in the DEER modulation depth of less than ±2.5%, as found previously (Georgieva, Borbat et al. 2015). Data are shown for spin-labeled L46C residue in DOPC/POPS-reconstituted $M2TMD_{21-49}$ at P/L's of 1:160 with amantadine, and 1:1,300 with and without amantadine.

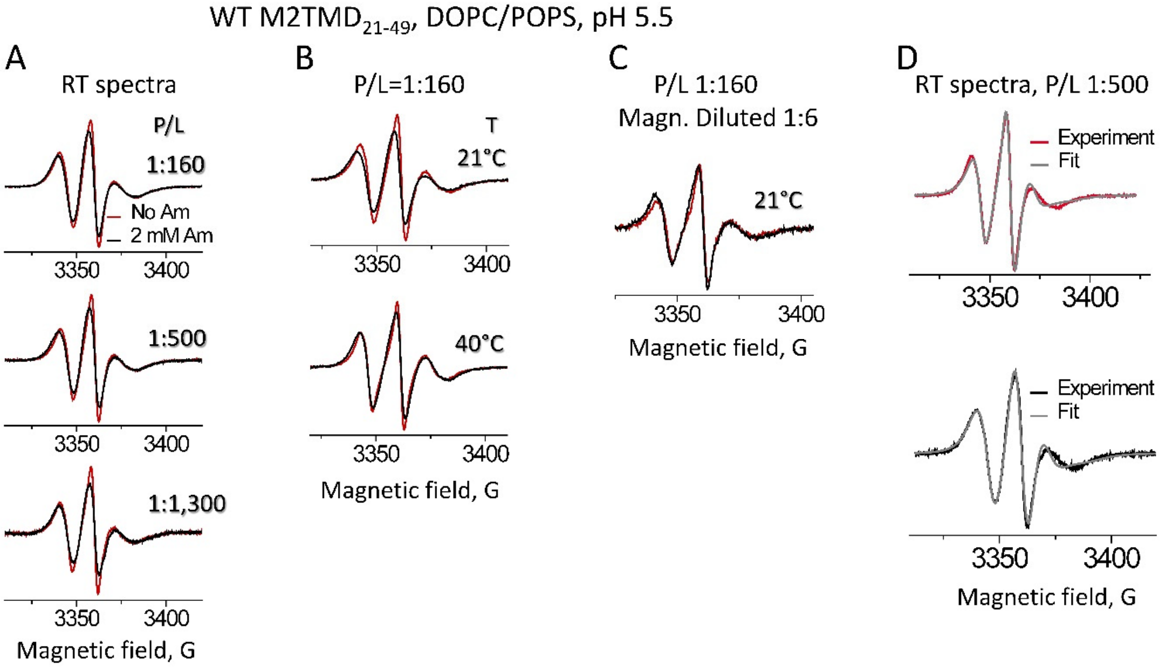

Supplementary Figure 3. The X-band (ca. 9.4 GHz) cw ESR spectra of wt $M2TMD_{21\text{-}49}$, spin-labeled at position L46C are shown. Data with and without amantadine are drawn in black and red colors, respectively. **A)** Spectra recorded at room temperature (ca. 25 °C) for $M2TMD_{21\text{-}49}$ in DOPC/POPS for P/L's 1:160, 1:500 and 1:1,300. **B)** The spectra for P/L of 1:160 at controlled temperatures of 21 ºC and 40 ºC. **C)** ESR spectra recorded at 21 ºC on $M2TMD_{21\text{-}49}$ magnetically diluted by the 1:6 ratio of spin-labeled to unlabeled peptide. The cw ESR lineshape broadening occurs upon the addition of amantadine, indicating drug-induced change in the spin-label motional dynamics. This broadening persists even in the spectra recorded at 40 ºC and also in the magnetically diluted sample, (i.e. it is unrelated to dipole-dipole coupling). **D)** To better understand these effects, we applied the MOMD NLLS spectral fitting (Budil, Lee et al. 1996) to the RT spectra for P/L's of 1:500 and 1:1,300 to determine the rotational correlation time, τ_c and the order parameter, S_{20}. In all cases, we obtained the best fit for τ_c = 2.3 ns. However, S_{20} increased for the samples with amantadine: S_{20} = 0.11 for apo vs. S_{20} = 0.18 for the drug-bound $M2TMD_{21\text{-}49}$ for both P/L's of 1:1,300 and 1:500, suggesting more space restricted spin-label motion was imposed by amantadine-induced structural changes.

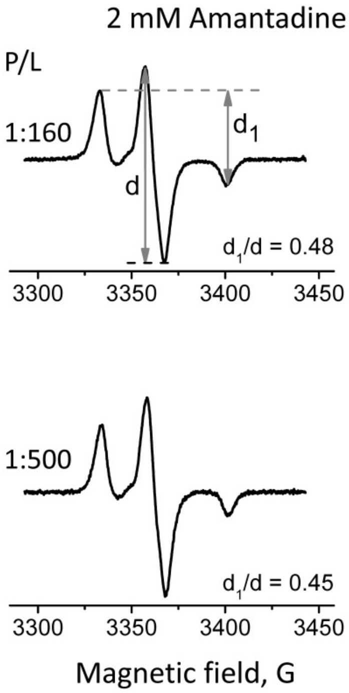

Supplementary Figure 4. Low temperature (163 K) X-band (ca. 9.4 GHz) cw ESR spectra of spin-labeled L46C residue for amantadine-bound $M2TMD_{21-49}$ in DOPC/POPS membranes. The d_1/d parameter was used to test the presence of inter-spin distances less than 2 nm (Kokorin 2012, p. 113-164). The inter-spin distance of 1.89 nm corresponds to d_1/d of 0.54, whereas smaller d_1/d values correspond to distances longer than 2 nm. Here, for amantadine-bound $M2TMD_{21-49}$ we obtained d_1/d parameter less than 0.48, indicating that the distances are well within the range of DEER method, i.e. 1.7 nm. This fact also holds for amantadine-free $M2TMD_{21-49}$, since in the previous study we found $d_1/d \leq 0.51$; the conclusion was further reinforced by double-quantum coherence ESR (DQC) conducted on magnetically diluted sample, showing a negligible content of distances shorter than 1.7 nm (Georgieva, Borbat et al. 2015).

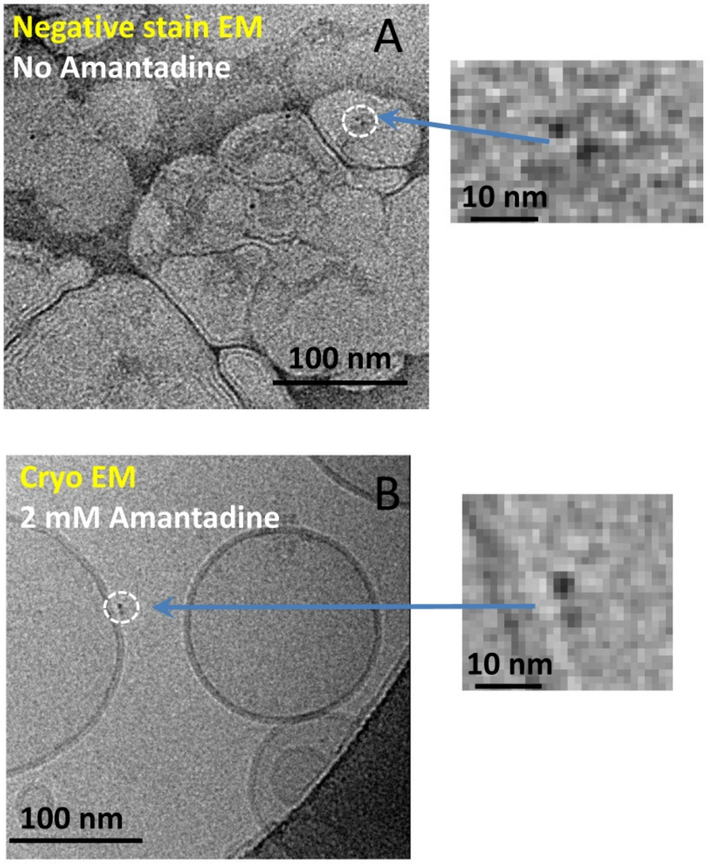

Supplementary Figure 5. EM micrographs of $M2TMD_{21-49}$ labeled with gold nanoparticles at position L46C and reconstituted into DOPC/POPS membranes. EM micrographs, both negative stain TEM shown in panel **A** and cryoEM shown in panel **B** demonstrate very high resolution.

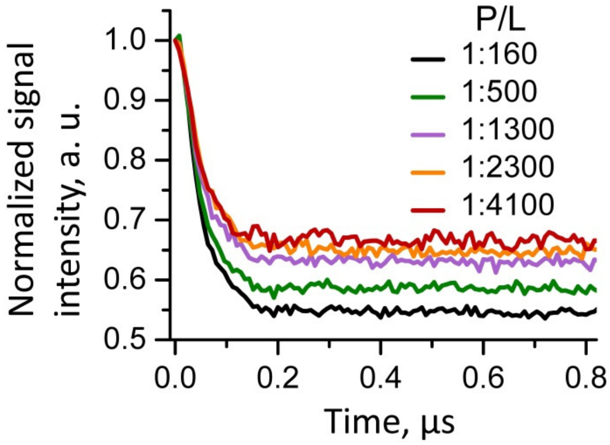

Supplementary Figure 6. The background-corrected and normalized to unity at zero time DEER data from spin-labeled $M2TMD_{21-49}$ in DOPC/POPS with the addition of 2 mM amantadine are plotted for several P/L's of 1:4,100, 1:2,300, 1:1,300, 1:500, and 1:160. The DEER modulation depth increases monotonically with P/L. Only the early 0.8 μs of the DEER data are shown for clarity.

Supplementary references

Budil, D. E., S. Lee, S. Saxena and J. H. Freed. "Nonlinear-least-squares analysis of slow-motion EPR spectra in one and two dimentions using a modified Levenberg-Marquard algorithm." *Journal of Magnetic Resonance* (1996) **120**: 155-189.

Georgieva, E. R., P. P. Borbat, H. D. Norman and J. H. Freed. "Mechanism of influenza A M2 transmembrane domain assembly in lipid membranes." *Sci Rep* (2015) **5**: 11757.

Kokorin, A. I. (2012, p. 113-164). Forty years of the d1/d parameter. A. I. Kokorin, InTech.
